# Clinical and Epidemiological Features of Tuberculous Pleural Effusion in Alicante, Spain

**DOI:** 10.3390/jcm10194392

**Published:** 2021-09-26

**Authors:** Eusebi Chiner, Miriam Nomdedeu, Sandra Vañes, Esther Pastor, Violeta Esteban, Carmen Castelló, Ignacio Boira, Virginia Molina, Juan M. Arriero, Jose N. Sancho-Chust

**Affiliations:** Secció de Pneumologia, Hospital Universitari Sant Joan d’Alacant, Ctra Alacant-València s/n, Sant Joan d’Alacant, 03550 Alicante, Spain; MiriamNmdfdz@gmail.com (M.N.); sandravanesbanos@gmail.com (S.V.); epastorespla@gmail.com (E.P.); Violeta_ER@hotmail.com (V.E.); carmencfaus@gmail.com (C.C.); nachoboiraenrique@hotmail.es (I.B.); virginia_molpe@hotmail.com (V.M.); juanmarriero@hotmail.com (J.M.A.); norbertosancho@gmail.com (J.N.S.-C.)

**Keywords:** tuberculous pleural effusion, immigrant population, thoracentesis, pleural biopsy

## Abstract

We aimed to (1) evaluate the incidence of tuberculous pleural effusion (TPE) over 25 years in our centre; (2) measure the yield of different diagnostic techniques; (3) compare TPE features between immigrant and native patients. Retrospective study of patients who underwent diagnostic thoracentesis and pleural biopsy in our hospital between 1995 and 2020. TPE was diagnosed in 71 patients (65% natives, 35% immigrants). Onset was acute in 35%, subacute in 26% and prolonged in 39%. Radiological features were atypical in 42%. Thoracentesis specimens were lymphocyte-predominant in 84.5% of patients, with elevated adenosine deaminase in 75% of patients. Diagnostic yield of pleural biopsy was 78%. Compared with native patients, more immigrants had previous contact with TB (54% vs. 17%, *p* = 0.001), prior TB (21% vs. 4%, *p* < 0.02) and atypical radiological features (58% vs. 34%, *p* < 0.03). TPE incidence was six times higher in the immigrant population (6.7 vs. 1.1 per 100,000 person-years, *p* < 0.001). TPE has an acute onset and sometimes atypical radiological features. Pleural biopsy has the highest diagnostic yield. Reactivation, prior contact with TB, atypical radiological features, complications, and positive microbiology results are more common in immigrant patients.

## 1. Introduction

Extrapulmonary Tuberculosis (TB) accounts for 15% of reported cases, tuberculous pleural effusion (TPE) being the most common form [1]. In industrialised countries, epidemiological patterns have changed, with a decrease in new cases among the native population and an increase among immigrants [2].

TPE occurs in both primary infection and reactivation [3]. The mean annual prevalence of pleural TB is 3–5% in areas with low TB prevalence [4], and up to 30% in endemic areas, owing partly to the larger proportion of human immunodeficiency virus (HIV)-positive inhabitants [5].

TPE occurs when a subpleural caseous focus ruptures and mycobacterial antigens enter the pleural space [6]. In the parietal pleura, lymphatic stomata can become blocked by the inflammation, leading to an accumulation of pleural fluid [7]. TPE mostly affects young people, although in non-endemic areas, where disease reactivation is frequent, older people are also affected [8].

The symptoms of this entity vary considerably [9]. Acute onset is more common in young people and atypical features are more common in HIV+ patients [10,11].

We hypothesise that the TPE population treated in our regional Department of Health (Alicante) has changed over the years with migration flows, and that the clinical and epidemiological features of the disease may vary between the immigrant and native population. This study aims to (1) determine the incidence of TPE in a large series spanning 25 years; (2) assess the yield of different diagnostic techniques; (3) assess the clinical and radiological features of TPE and compare these variables in immigrant and native patients.

## 2. Materials and Methods

### 2.1. Type and Period of Study

This is a retrospective cohort study based on a database created for the Pulmonology Department of Sant Joan d’Alacant University Hospital (catchment population: 240,000 inhabitants) in 1995 including demographic and clinical characteristics of patients undergoing diagnostic thoracentesis and pleural biopsy. The study period spanned from January 1995 to March 2020.

### 2.2. Inclusion and Exclusion Criteria

Patients with pleural effusion who underwent diagnostic thoracentesis and percutaneous closed needle pleural biopsy were included. Light criteria were used to differentiate exudative from transudative pleural effusion [12]. Patients with altered level of consciousness or clotting disorders, and those in whom samples could not be taken, were excluded.

### 2.3. Definition of Populations

The native population was defined as people born and residing in Spain, while the immigrant population was defined as people born outside Spain and residing in Spain [13].

### 2.4. Diagnostic Delay

We studied total delay, defined as the time with symptoms until diagnosis and treatment; and patient-attributable delay, defined as the time with symptoms before the patient sought medical assistance. Total diagnostic delay was defined as unacceptable using a cutoff value of a maximum of 4 weeks [14].

### 2.5. Case Definition

TPE diagnoses had to meet at least one of the following criteria: (1) isolation of mycobacteria in sputum or in bronchoscopy, thoracentesis or pleural biopsy specimens; (2) presence of granuloma in pleural biopsy specimen; (3) exudative lymphocytic pleural effusion and adenosine deaminase (ADA) levels ≥ 40 U/L in pleural fluid; (4) exudative lymphocytic pleural effusion and pleural biopsy showing pleurisy in absence of malignancy; (5) lymphocytic effusion resolved with empiric tuberculosis treatment in patients with high probability but negative microbiology and pathology results.

### 2.6. Procedures

Samples from thoracentesis were sent under anaerobic conditions for pH testing, biochemistry, cell count, microbiology, and cytology.

Biochemical parameters included proteins, glucose, lactate dehydrogenase, ADA, carcinoembryonic antigen, albumin, and cholesterol [7]. Other diagnostic techniques included Gram stain, Ziehl-Neelsen stain, culture on aerobic and anaerobic media, culture on Löwenstein-Jensen medium, culture in liquid medium, and, in recent years, newer tests such as rapid immunochromatographic detection of the MPT64 antigen.

For the pleural biopsy, a minimum of three samples were taken: one for pathology and two or three for microbiology (Ziehl-Neelsen stain and culture on Löwenstein-Jensen medium). Specific closed pleural biopsy needles were used, mainly Abrams’ needle but also Cope’s and Ramel’s needles in some cases. Over the study period, thoracic ultrasound was introduced to guide the procedure.

Diagnostic bronchoscopy was performed at the discretion of a doctor when imaging tests showed a parenchymal lesion.

### 2.7. Statistical Analysis

The variables collected were: age; sex; country of birth; year of diagnosis; smoking status; pack-years; contact with TB; prior TB; predisposing factors (immunodeficiency, use of steroids, HIV infection, use of biologics); comorbidities; radiographic features and extent (mild: less than lower third; moderate: less than half; massive: more than two thirds; atypical: not free-flowing); area occupied by pleural effusion; CT (yes/no); bronchoscopy (yes/no); involvement of adjacent parenchyma; symptoms (cough, expectoration, haemoptysis, chest pain, fever, dyspnoea, constitutional symptoms); onset duration (acute: <1 week, subacute: <4 weeks; prolonged: ≥4 weeks); time with symptoms until admission; time from admission to thoracentesis and/or pleural biopsy and treatment; estimated patient-attributable delay time; total delay time; tuberculin skin test; pleural fluid biochemistry results; ADA test (with cutoffs of 30 and 40 U/L); appearance of pleural fluid; pleural fluid and pleural biopsy microbiology results; pleural biopsy pathology and cytology results; chest drainage; length of hospital stay; treatment prescribed; drug resistance; use of steroids; progress; radiological sequelae after 3 months and 1 year.

We performed a descriptive analysis of the quantitative and categorical variables. To compare quantitative variables between groups we used the unpaired Student *t*-test, or nonparametric tests in the case of unequal variances. For categorical variables, we used the Chi-square test or Fisher’s exact test. *p* values below 0.05 were considered significant. The statistical analysis was performed using SPSS version 18.0 (Chicago, IL, USA).

### 2.8. Ethical Principles

While conducting this study we followed the provisions laid down in the Declaration of Helsinki and updated in Edinburgh in 2000. The protocol was approved by the ethics committee of San Juan de Alicante University Hospital.

## 3. Results

In the study period, 3675 thoracenteses were performed in 2677 patients (mean age 68 ± 14 years). The pleural effusion was exudative in 2088 cases (78%): neoplastic (44%), parapneumonic (35%), pulmonary embolism (8%), another cause (5.5%), and idiopathic (4%). TPE was diagnosed in 71 patients (3.5% of exudative effusions): 48 men (68%), mean age 35 ± 14 years (range 15–94 years). The mean annual number of TPE diagnoses was 3, giving a global incidence rate of 1.25 per 100,000 person-years. The incidence in the native population was 1.1 per 100,000 person-years, and in the immigrant population, 6.7 per 100,000 person-years (*p* = 0.001).

**Figure 1:**
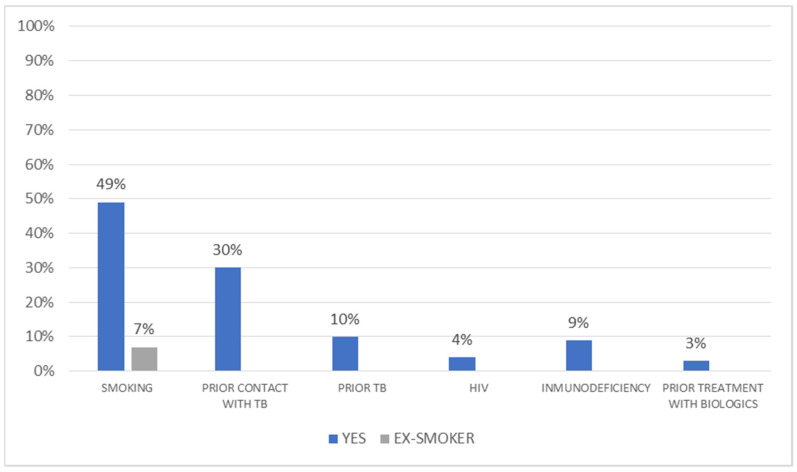
Risk factors for tuberculosis in the study population. TB: Tuberculosis.

Figure 1 shows the risk factors for TB. Regarding smoking status, 35 patients (49%) were current smokers, 31 (44%) were non-smokers, and 5 (7%) were ex-smokers. The mean number of pack-years was 18 ± 12. Comorbidities were recorded in 39% of patients.

**Figure 2:**
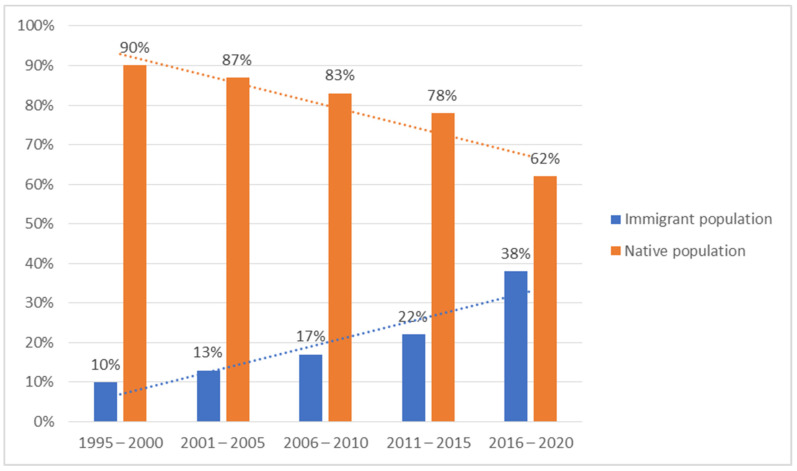
Proportion of native and immigrant patients over the study period.

Of all TPE patients, 46 (65%) were natives and 25 (35%) were immigrants: 10 from Latin America, 6 from Morocco, 3 from Senegal, 2 each from Argelia and Eastern Europe, and 1 each from Pakistan and Mauritania. Figure 2 shows the proportion of native and immigrant patients in 5 year periods.

Of all patients, 73% were referred from the Emergency Department, 17% by their GP and 10% by other specialists. Onset duration was acute in 35% of patients, subacute in 26% and prolonged in 39%. The most common symptom was pleuritic chest pain (83%), followed by fever (76%), cough (65%), dyspnoea (63%), constitutional symptoms (32%), purulent sputum (20%) and haemoptysis (1%).

The tuberculin test was positive in 38%. Atypical pleural effusion was recorded in 42%. The effusion was right-sided in 52% of cases, left-sided in 44%, bilateral in 4% and pleuropericardial in 3%. The extent of pleural effusion was mild in 34% of patients, moderate in 38% and massive in 28%. Thirty-two per cent of patients had parenchymal infiltration and 45% underwent CT scans, half of which provided diagnostic evidence.

There was a diagnostic delay in 69% of cases, with a mean patient-attributable delay of 31 ± 20 days (range 9–100) and a mean total delay of 36 ± 29 days (range 7–142). Table 1 displays the time to thoracentesis/biopsy, time to beginning of treatment, and length of hospital stay.

The pleural fluid appeared serous in 67% of patients, serosanguineous in 10%, cloudy in 17%, purulent in 3% and clear in 3%. Most effusions were lymphocytic (84.5%). With a cutoff point of 40 U/L, the ADA test gave a positive result in 75% of cases. At 30 U/L, the proportion of positive results increased to 84.5% (*n* = 60) (Table 2).

The diagnostic yield of microbiology in pleural fluid and pleural biopsy specimens is shown in Table 3 and Table 4. Rapid MPT64 antigen test (43 patients) had a positive result in 30%. Cytology of the pleural fluid showed inflammatory characteristics in 89% of cases and nonspecific characteristics in 11%. Pleural biopsy (58 patients) showed a positive diagnosis in 78% of these cases. Granulomas were found in 71% of pleural biopsy specimens, showing microbacteria-harboring macrophages at the centre surrounded by a rim of lymphocytes. Pleurisy was found in 22% of pleural biopsy specimens, showing non-specific chronic pleural inflammation. The remaining samples were nonspecific. In three patients (4%), diagnosis was based on resolution of pleural effusion with empiric TB therapy, as all test results had come back negative. Bronchoscopy (19 patients) provided diagnostic evidence in 4 cases (mycobacteria detected in bronchial aspirate by Ziehl-Neelsen stain (*n* = 1) or polymerase chain reaction (*n* = 1); compatible histology (*n* = 2)).

Regarding treatment, four drugs were prescribed in 83% of cases and three drugs in 17%. In three patients (4.2%), the TPE was resistant to isoniazid. Seven (9.9%) received a short course of steroids. Tube thoracostomy was performed in 28% of patients, in order to manage tuberculous empyema and symptomatic or complicated pleural effusions.

After three months, 58% of cases were completely resolved, 7% had minimal pleural effusion, 3% showed a reduction of more than 50%, and 32% had minimal pleural thickening. After 12 months, only thickening was observed (in 12% of cases) and all patients were considered cured.

Comparison between immigrant and native populations is shown in Table 5. In the immigrant population, cholesterol was lower (*p* = 0.04), lymphocyte predominance was higher (*p* = 0.02), and neutrophil-predominance was lower (*p* = 0.02). We found no significant differences between the populations in terms of age, sex, smoking status, comorbidities, or immunodeficiency.

The immigrant population was more likely to have prior contact with TB (54% vs. 17%, *p* = 0.001), and a prior history of TB (21% vs. 4%, *p* = 0.01). We found no significant differences in symptoms. Immigrants were more likely to show atypical radiological features such as organised pleural effusion, pyothorax or hydropneumothorax (58% vs. 34%, *p* = 0.03).

We found no significant differences between the groups in terms of the appearance of the pleural fluid, ADA levels greater than or equal to 30 U/L or 40 U/L, or lymphocytic predominance. The immigrant population had significantly more cultures in liquid medium (47.4% vs. 10.3%, *p* < 0.05), and nonsignificant trends included higher positivity in pleural fluid Ziehl-Neelsen stains (12.5% vs. 2.1%, *p* = 0.07) and fewer pleural biopsies (71% vs. 87%, *p* = 0.09). Regarding the contribution of histology of pleural biopsy, we found no differences between the groups.

The percentage of positive tuberculin tests was higher in the immigrant population (54% vs. 29.8%, *p* < 0.05). We found no differences between the groups in terms of length of hospital stay, treatment, drug resistance, use of steroids, or follow-up.

## 4. Discussion

In our study, TPE accounted for 3.5% of exudates, slightly less than in other series [15], and affected young people (mean age 35 years), as is typical. Two thirds of the population had no comorbidities and very few had classic risk factors such as immunodepression or HIV (4.2%), unlike in previous pulmonary TB series [16].

Although only one third of our TPE patients were immigrants, their proportion increased steadily during the study period. This trend is clearly related to the movement of populations [2,13,17].

Most patients had a typical clinical presentation, with a high proportion of acute onset [18]. Of concern were the patient-attributable and total diagnostic delays recorded in 69% of patients, with a mean delay of more than 30 days and a maximum of 142 days. Few studies have examined this aspect of TPE [17].

A high percentage of our series had atypical (42%) or moderate/severe pleural effusion (66%), and almost one third had parenchymal infiltrate (suggestive of reactivation), with CT scans improving diagnostic sensitivity [19]. As in other series, two thirds of our patients had a negative tuberculin test, suggesting that they were tested during the non-reactive stage of a primary infection.

Neutrophil-predominance was found in 15.5% of patients, probably reflecting an initial stage of pleural compromise [20]. When this occurs, Mycobacterium tuberculosis may be more easily isolated in pleural fluid or sputum [21]. In our series, three quarters of patients had ADA levels of 40 U/L or above. Other authors have applied a 30 U/L cutoff point [22], which in our study would have increased sensitivity to 85%.

In Spain, the incidence of TB is gradually decreasing, with the rate of extrapulmonary forms varying with age (between 3 and 10 cases per 100,000 person-years). In our department, the incidence of extrapulmonary TB was 6.37 per 100,000 person-years, similar to the figure reported for the Valencian community, which is currently among the lowest in Spain [23].

Since ADA testing can give false positives [24], it must be combined with microbiological and histological techniques in pleural fluid. Pleural biopsy provides the highest diagnostic yield, varying from 51 to 88% in the literature [7,15], being relatively high in our study.

Some differences were found between immigrant and native patients. Immigrants were more likely to have prior contact with TB and a prior history of TB, meaning the infection was more likely to be a reactivation. In addition, immigrants showed more atypical radiological features. The higher proportion of prior TB and pyothorax may explain the higher positivity in pleural fluid Ziehl-Neelsen stains and liquid-medium cultures among immigrants. The higher proportion of positive tuberculin tests in immigrants may be due to the greater proportion of reactivation, prior contact and possibly the longer duration of infection, which may have exceeded the window period. Finally, higher percentage of lymphocytic pleural effusion was found in the immigrant population, but no difference in the proportion of ADA levels greater than or equal to 30 U/L or 40 U/L. This contradicts the findings of previous studies that showed an association between lymphocytosis and positive ADA test results [25].

Our study has some potential limitations, such as its retrospective nature and our initial ignorance of the type of immigrant patients we would be seeing over the years, which limited our ability to make precise comparisons. However, the long study period and centralised data on TB in our pulmonology department enabled us to confirm that both populations were representative and sufficiently large for inclusion in a global assessment of the clinical and epidemiological characteristics of TPE.

The phenomenon of immigration has changed the epidemiological pattern of PTE in western countries. Although the overall incidence of TPE in our study was relatively low, the incidence among immigrants was six times higher than among native patients. Reactivation, prior contact with TB, atypical radiological features, complications, and positive microbiology results were more common in immigrant patients. Clinical presentation and diagnostic delay were similar in the two groups, and all patients were completely cured after one year with no significant sequelae.

## 5. Conclusions

TPE is an entity that mainly affects young people and generally has an acute onset and few predisposing factors. Radiological findings are often atypical, but post-treatment sequelae are rare. In two thirds of cases, diagnosis is based on a combination of lymphocytic pleural fluid with ADA levels greater than or equal to 40 U/L, although pleural biopsy has a higher diagnostic yield. Diagnostic delay is very frequent.

## Figures and Tables

**Table 1:** Time from hospital admission to diagnostic tests, to beginning of treatment, and to discharge.

Time (Days)	Mean	SD
Thoracentesis	3	2
Pleural biopsy	4	2
Treatment	5	3
Discharge	12	9

SD: standard deviation.

**Table 2:** Biochemical characteristics of pleural fluid in patients with tuberculous pleural effusion.

Parameter	Mean	SD
pH	7.29	0.10
Glucose (mg/dL)	72.51	29.07
Proteins (g/dL)	4.94	0.72
LDH (U/L)	668	500
Cholesterol (mg/dL)	84.83	22.42
Pleural fluid to serum protein ratio	0.74	0.08
Pleural fluid to serum LDH ratio	1.09	0.49
ADA (U/L)	54	23
CEA (ng/mL)	2.11	2.25
Erythrocytes (per mm^3^)	6230	8079
Leukocytes (per mm^3^)	3760	4352
Lymphocytes (%)	74	25
Neutrophil (%)	25	25

SD: standard deviation; LDH: lactate dehydrogenase; ADA: adenosine deaminase; CEA: carcinoembryonic antigen.

**Table 3:** Diagnostic yield of microbiology tests in pleural fluid of patients with tuberculous pleural effusion.

	Positive	Negative
Ziehl-Neelsen stain	4 (6%)	67 (94%)
Culture in solid medium	18 (25%)	53 (75%)
Culture in liquid medium	12 (25%)	36 (75%
MPT64 antigen detection	13 (30%)	30 (70%)

**Table 4:** Diagnostic yield of microbiology tests in pleural biopsy specimens from patients with tuberculous pleural effusion.

	Positive	Negative
Ziehl-Neelsen stain	8 (14%)	50 (86%)
Culture in solid medium	23 (40%)	35 (60%)
Culture in liquid medium	9 (23%)	30 (77%)

**Table 5:** Comparison of diagnostic delay times and biochemical characteristics of tuberculous pleural effusion in the immigrant and native population.

	Immigrant (Mean ± SD)	Native (Mean ± SD)	*p* Value
Age	35 ± 12	35 ± 14	ns
Total diagnostic delay (days)	38 ± 27	34 ± 30	ns
Estimated duration of disease (days)	33 ± 27	29 ± 28	ns
Days from admission to biopsy	4 ± 1	4 ± 2	ns
Days from admission to thoracentesis	3 ± 1	3 ± 2	ns
Days from admission to treatment	5 ± 2	6 ± 4	ns
Length of hospital stay (days)	12 ± 9	11 ± 9	ns
pH	7.29 ± 0.07	7.30 ± 0.12	ns
Glucose (mg/dL)	75 ± 34	71 ± 26	ns
Proteins (g/mL)	5 ± 0.8	4.8 ± 0.7	ns
LDH (U/L)	640 ± 485	682 ± 512	ns
Cholesterol (mg/dL)	77 ± 18	89 ± 23	0.04
Pleural fluid to serum protein ratio	0.7 ± 0.1	0.7 ± 0.1	ns
Pleural fluid to serum LDH ratio	1.1 ± 0.6	1.1 ± 0.4	ns
ADA (U/L)	54 ± 23	54 ± 24	ns
Proportion of ADA ≥ 30 U/L	88 ± 34	83 ± 38	ns
Proportion of ADA ≥ 40 U/L	79 ± 41	77 ± 43	ns
Lymphocytes (%)	85 ± 18	69 ± 27	0.02
Neutrophils (%)	15 ± 18	31 ± 27	0.02

SD: standard deviation; LDH: lactate dehydrogenase; ADA: adenosine deaminase.

## Data Availability

Data is available in Respiratory Department of Sant Joan d’Alacant University Hospital.
